# Supplementary material for: TGS1 mediates 2,2,7-trimethyl guanosine capping of the human telomerase RNA to direct telomerase dependent telomere maintenance
Source: Nat Commun. 2022 Apr 28;13:2302. doi: 10.1038/s41467-022-29907-z (PMC9050681; doi:10.1038/s41467-022-29907-z)
Supplement: Supplementary file 1 — Supplementary Information [file 41467_2022_29907_MOESM1_ESM.pdf]

SUPPLEMENTARY INFORMATION

TGS1 mediates 2,2,7-trimethyl guanosine capping of the human telomerase RNA to direct telomerase dependent telomere maintenance

Valentina Buemi[1,#,†], Odessa Schillaci[1,#], Mariangela Santorsola[1], Deborah Bonazza[2], Pamela Veneziano Broccia[1], Annie Zappone[1], Cristina Bottin[3], Giulia Dell'Omo[5], Sylvie Kengne[1], Stefano Cacchione[4], Grazia Daniela Raffa[4], Silvano Piazza[6], Fabrizio d'Adda di Fagagna[5,7], Roberta Benetti[8], Maurizio Cortale[9], Fabrizio Zanconati[2,3], Giannino Del Sal[1,5,6], Stefan Schoeftner[1,*]

1. Dipartimento di Scienze della Vita, Università degli Studi di Trieste, Via E. Weiss 2, 34127 Trieste, Italy

2. Struttura Complessa di Anatomia ed Istologia Patologica, Azienda Sanitaria Universitaria Giuliano Isontina (ASUGI) Trieste, Strada di Fiume 447, 34149 Trieste, Italy.

3. Dipartimento Universitario Clinico di Scienze Mediche Chirurgiche e della Salute, Università degli Studi di Trieste, Ospedale di Cattinara - Strada di Fiume 447 - 34149 Trieste, Italy.

4. Dipartimento di Biologia e Biotecnologie, Sapienza Università di Roma, Roma, Italy.

5. IFOM Foundation-FIRC Institute of Molecular Oncology Foundation, Milan 20139, Italy

6. International Centre for Genetic Engineering and Biotechnology (ICGEB), Area Science Park - Padriciano, 34149 Trieste, Italy

7. Istituto di Genetica Molecolare, Consiglio Nazionale delle Ricerche (IGM-CNR), Pavia 27100, Italy

8. Dipartimento di Area Medica (Dame), Università degli Studi di Udine, p.le Kolbe 4, 33100 Udine, Italy

9. Struttura Complessa di Chirurgia Toracica, Azienda Sanitaria Universitaria Giuliano Isontina (ASUGI) Trieste, Strada di Fiume 447, 34149 Trieste, Italy.

# contributed equally to the manuscript;

[†] current address: Cancer Research UK Cancer Therapeutics Unit, The Institute of Cancer Research, London, SM2 5NG, United Kingdom

*Corresponding author: e-mail: sschoeftner@units.it

CONTENT:

Supplementary figures 1-7

Supplementary tables 1-3

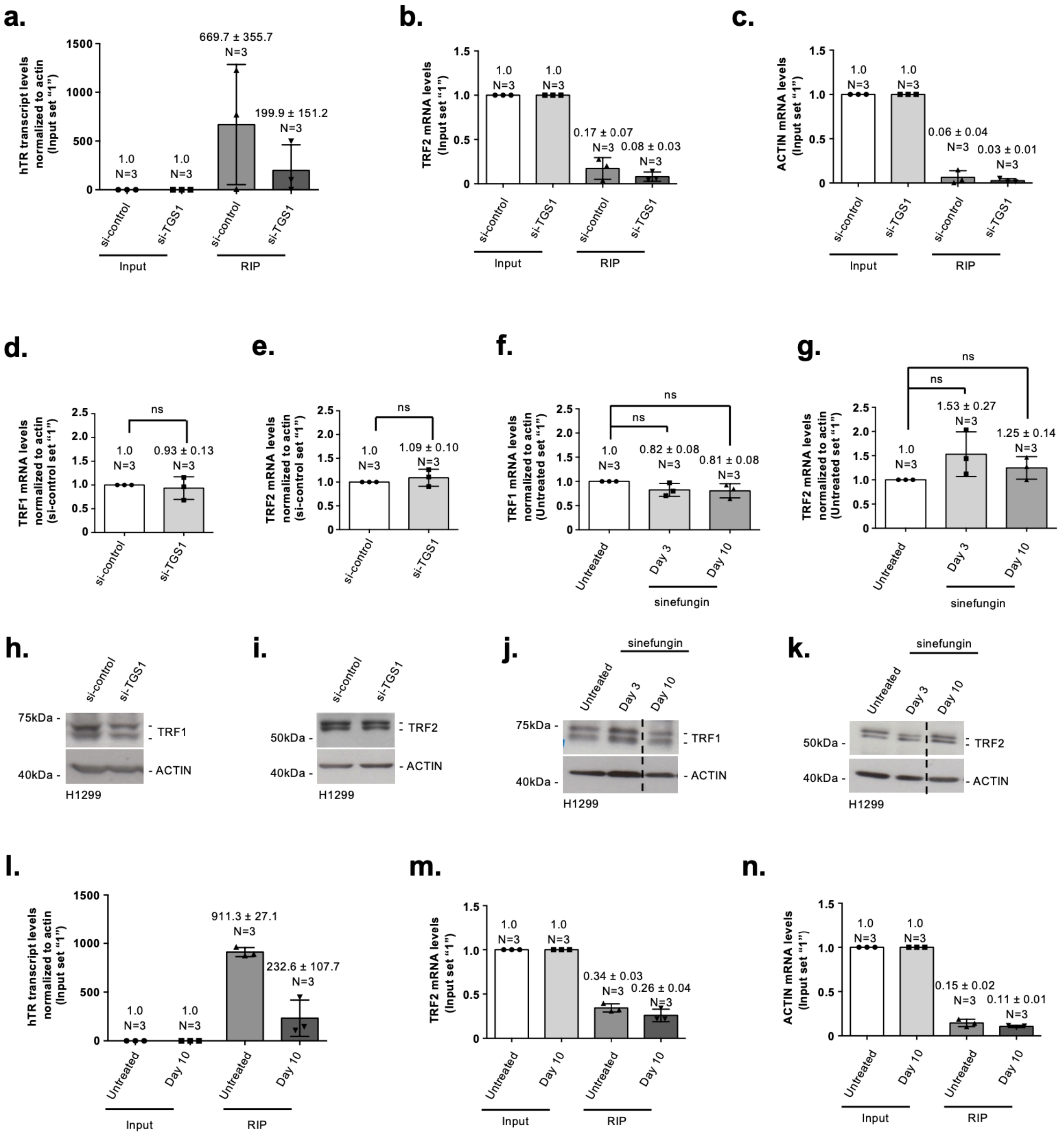

Supplementary Figure 1. TGS1 loss of function impairs 2,2,7-trimethyl guanosine capping of the human telomerase RNA.

a-c. Quantification of hTR, TRF2 and actin transcript enrichment compared to the input after RNA immunoprecipitation using specific anti-TMG antibody followed by qRT-PCR using lysates from H1299 cells transfected with the indicated siRNAs. **d, e**. Quantification of TRF1 and TRF2 mRNA levels by qRT-PCR in H1299 cells transfected with the indicated siRNAs. Values were normalized against actin. **f, g**. Quantification of TRF1 and TRF2 mRNA levels by qRT-PCR in H1299 cells treated with sinefungin for 3 and 10 days. Values were normalized against actin. **h, i**. Western blotting of protein extracts derived from H1299 cells transfected with the indicated siRNAs. **j, k**. Western blotting of protein extracts derived from H1299 cells untreated or treated with sinefungin for 3 and 10 days. **l-n**. Quantification of hTR, TRF2 and actin transcript enrichment compared to the input after RNA immunoprecipitation using specific anti-TMG antibody followed by qRT-PCR using lysates from H1299 cells treated or untreated with sinefungin for 10 days.

For quantifications in a – g and l - n mean values are indicated; whiskers indicate standard deviation. Experiments in panels h-k were repeated three times and gave reproducible results. N=number of independent experiments. A two-tailed Student's t-test was used to calculate statistical significance; p-values are shown. Uncropped blots in Source Data.

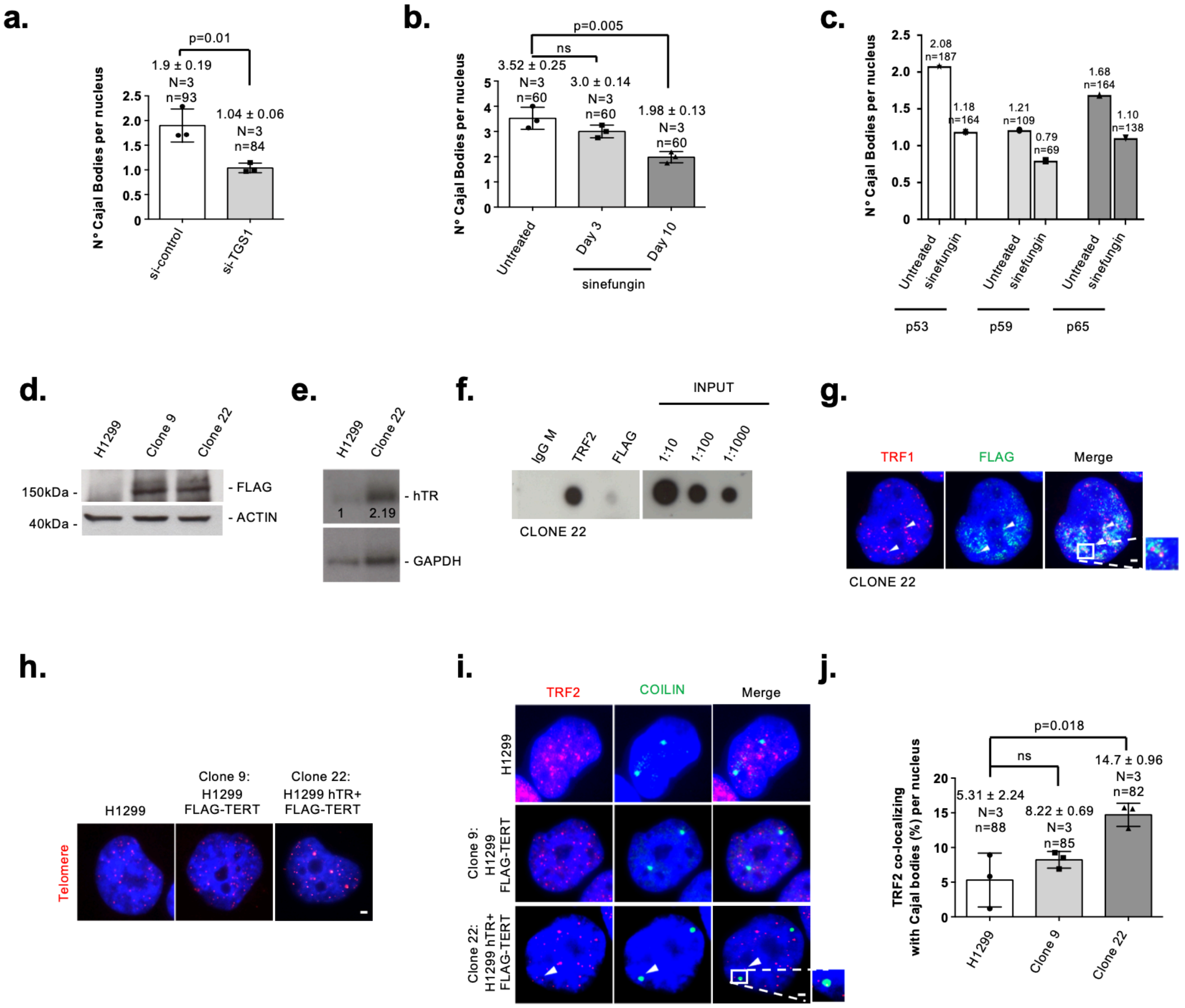

Supplementary Figure 2. Interaction of telomeres with Cajal bodies in H1299 cells overexpressing hTR and hTERT.

a. Number of Cajal bodies per nucleus in H1299 cells transfected with the indicated siRNAs. **b.** Number of Cajal bodies per nucleus in H1299 cells untreated or treated with sinefungin for 3 and 10 days. **c.** Number of Cajal bodies per nucleus in lung tumor organoids untreated or treated with sinefungin for 10 days. **d.** Western blotting showing expression of FLAG-tagged hTERT. Actin was used as loading control. **e.** Northern blotting of hTR levels showing expression of hTR in H1299 and clone 22 cells. A GAPDH probe was used as loading control. Values of quantitative densitometric analysis are shown. Expression levels in parental H1299 cells was set to "1". **f.** Chromatin immunoprecipitation experiments (ChIP) using clone 22 cells. Mouse anti-TRF2 and mouse anti-FLAG antibodies were used. Mouse control (IgM) was used as negative control. Serial dilutions of chromatin extract (input) prepared from clone 22 cells were loaded. **g.** Representative images of combined immunofluorescence using anti-TRF1 and anti-FLAG antibodies in clone 22 cells. **h.** Representative images of telomere DNA-FISH on interphase H1299 or clone 9 or clone 22 cells overexpressing FLAG-TERT or hTR/FLAG-TERT, respectively. **i.** Representative images of combined immunofluorescence using anti-TRF2 and anti-Coilin antibodies in H1299, clone 9 and clone 22 cells. **j.** Percentage of Cajal bodies co-localizing with telomeres (TRF2) per nucleus. For quantifications in a, b, c, j mean values are indicated; whiskers indicate standard deviation. Experiments in panels d-e were repeated three times and gave reproducible results. N=number of independent experiments. n=number of analyzed nuclei. Arrowheads indicate co-localization events. Red and green, epitopes detected by immunofluorescence; blue, DAPI stained nuclei. Scale bars 1 µm. A two-tailed Student's t-test was used to calculate statistical significance; p-values are shown. Uncropped blots in Source Data.

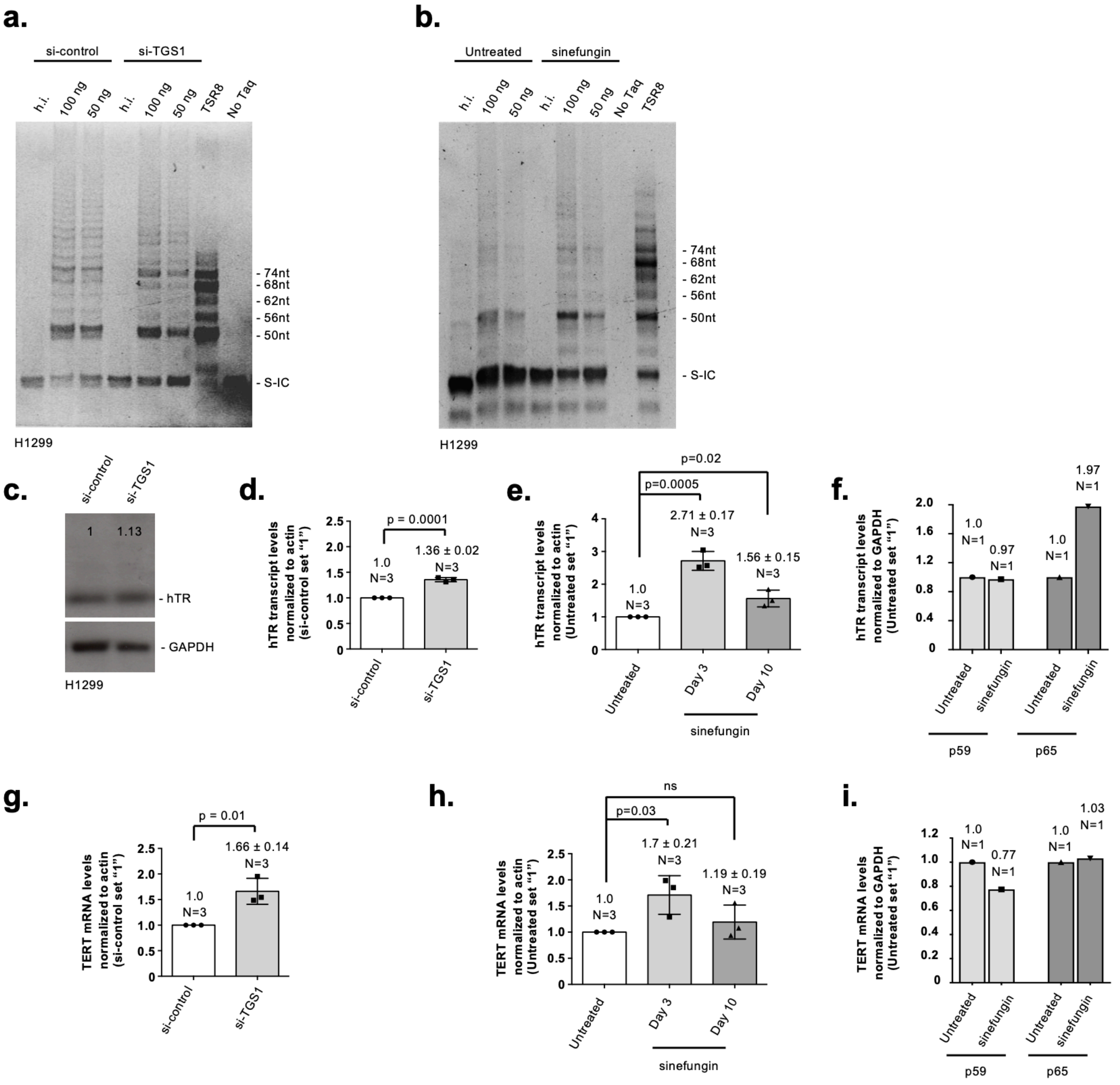

Supplementary Figure legend 3. Loss of TGS1 leads to elevated *in vitro* telomerase activity.

a. Telomerase activity in lysates of H1299 cells transfected with the indicated siRNAs for 10 days, as determined by the telomere repeat amplification protocol (TRAP). Numbers (100, 50) indicate the amount of protein lysate used for the assay (ng). **b.** Telomerase activity in lysates of H1299 cells treated with sinefungin for 10 days, as determined by the telomere repeat amplification protocol (TRAP). Numbers (100, 50) indicate the amount of protein lysate used for the assay (ng). **c.** Northern blotting of hTR levels in H1299 cells transfected with indicated siRNAs. GAPDH probe was used as loading control. Values of quantitative densitometric analysis are shown. si-control was set to "1". **d.** Quantification of hTR transcript levels by qRT-PCR using total RNA from H1299 cells repeatedly transfected for 10 days with the indicated siRNAs. **e.** Quantification of hTR transcript levels by qRT-PCR using lysates from H1299 cells untreated or treated for 3 and 10 days with sinefungin. **f.** Quantification of hTR transcript levels by qRT-PCR using lysates from lung tumor organoids untreated or treated for 10 days with sinefungin. **g.** Quantification of hTERT mRNA levels by qRT-PCR using total RNA from H1299 cells transfected for 10 days with the indicated siRNAs. **h.** Quantification of hTERT mRNA levels by qRT-PCR using total RNA from H1299 cells untreated or treated for 3 and 10 days with sinefungin. **i.** Quantification of TERT mRNA levels by quantitative RT-PCR using total RNA from lung tumor organoids untreated or treated for 10 days with sinefungin. h.i., heat inactivated; S-IC, internal control PCR product; TSR8 control template for PCR. For quantifications in panels d, e, f, g, h mean values are indicated; whiskers indicate standard deviation. Three independent experiments in panels a-c gave reproducible results. N=number of independent experiments. A two-tailed Student's t-test was used to calculate statistical significance; p-values are shown. Uncropped blots in Source Data.

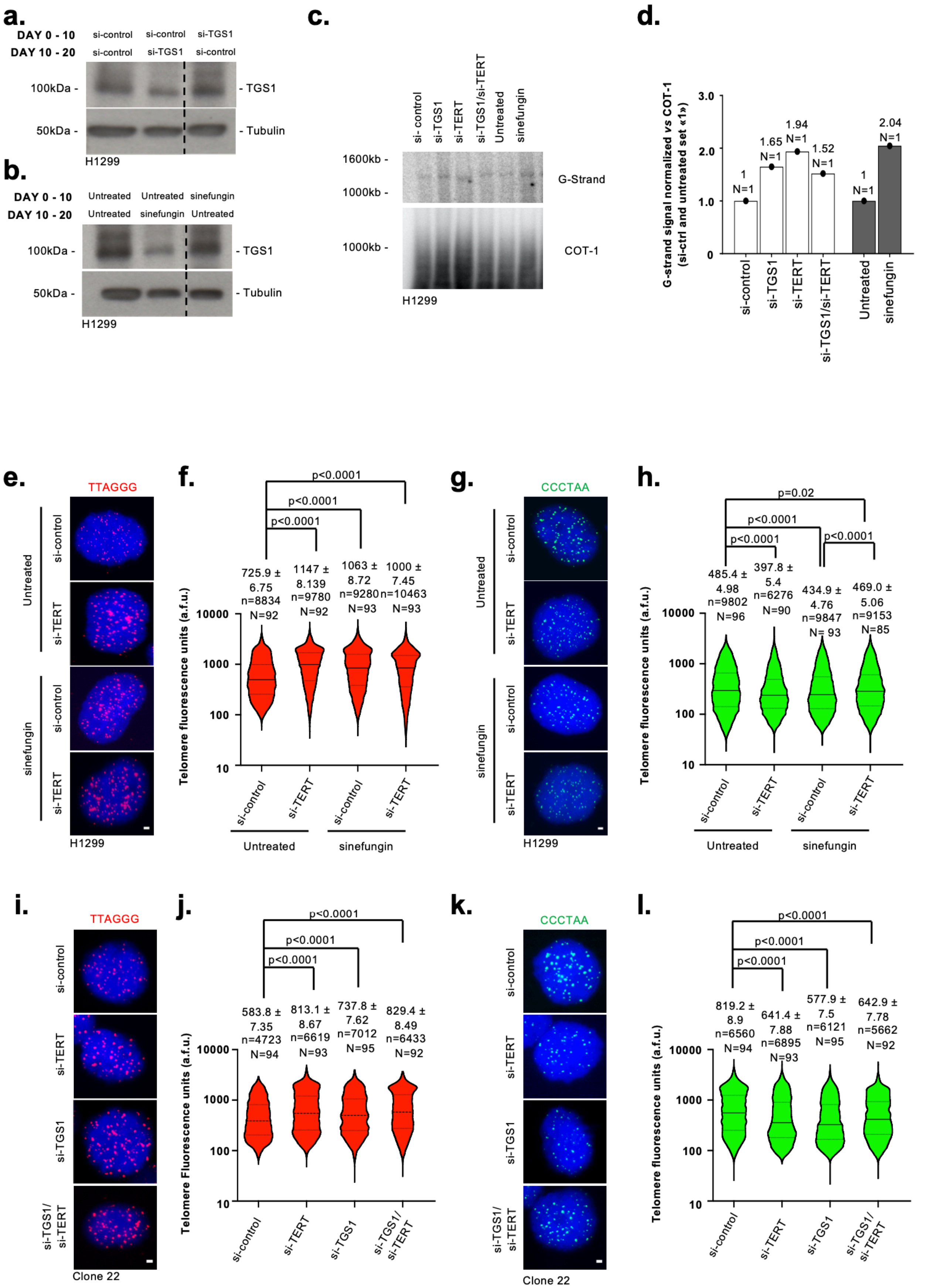

Supplementary Figure 4. Impaired TGS1 function leads to altered processing of telomere strands.
a, b. Western blotting of lysates from H1299 cells repeatedly transfected with indicated siRNAs, untreated or treated with sinefungin during a 20-day treatment scheme. Tubulin was used as loading control. Results

shown were obtained from single western blotting membranes. **c**. G-strand overhang analysis in H1299 transfected with indicated siRNAs, untreated or treated with sinefungin for 10 days. Bands show amount of G-strand overhangs. COT-1 DNA probe was used as loading control. **d.** Values of quantitative densitometric analysis of data shown in Suppl. Fig. 4c. **e.** Representative images of DNA-FISH experiments using a G-rich telomere strand (red) specific probe on H1299 cells repeatedly transfected with indicated siRNAs. When indicated, cells were treated with sinefungin. **f.** Quantification of G-rich telomere strand fluorescence intensity of cells shown in Suppl. Fig. 4e. **g.** Representative images of DNA-FISH experiments using a C-rich telomere strand (green) specific probe on interphase H1299 cells repeatedly transfected with indicated siRNAs. When indicated, cells were treated with sinefungin. **h.** Quantification of C-rich telomere strand fluorescence intensity of cells shown in Suppl. Fig. 4g. **i.** Representative images of DNA-FISH experiments using a G-rich strand telomere (red) specific probe on clone 22 cells repeatedly transfected with indicated siRNAs. **j.** Quantification of G-rich telomere strand fluorescence intensity of cells in Suppl. figure 4i. **k.** Representative images of DNA-FISH experiments using a C-rich strand telomere (green) specific probe on clone 22 cells repeatedly transfected with indicated siRNAs. **l.** Quantification of C-rich telomere strand fluorescence intensity of cells shown in Suppl. figure 4k. Experiments in Suppl. figure 4a-c were repeated twice. Panels f, h, j, l, middle line represents median arbitrary fluorescence units (a.f.u.), dotted lines mark highest and lowest quartiles. Mean values with standard deviation are indicated; N=number of analyzed nuclei, n=number of telomere repeat signals. Scale bars 1 µm. Red and green, telomere strand specific DNA FISH probes; blue, DAPI stained nuclei A two-tailed Student's t-test was used to calculate statistical significance; p-values are shown. Uncropped blots in Source Data.

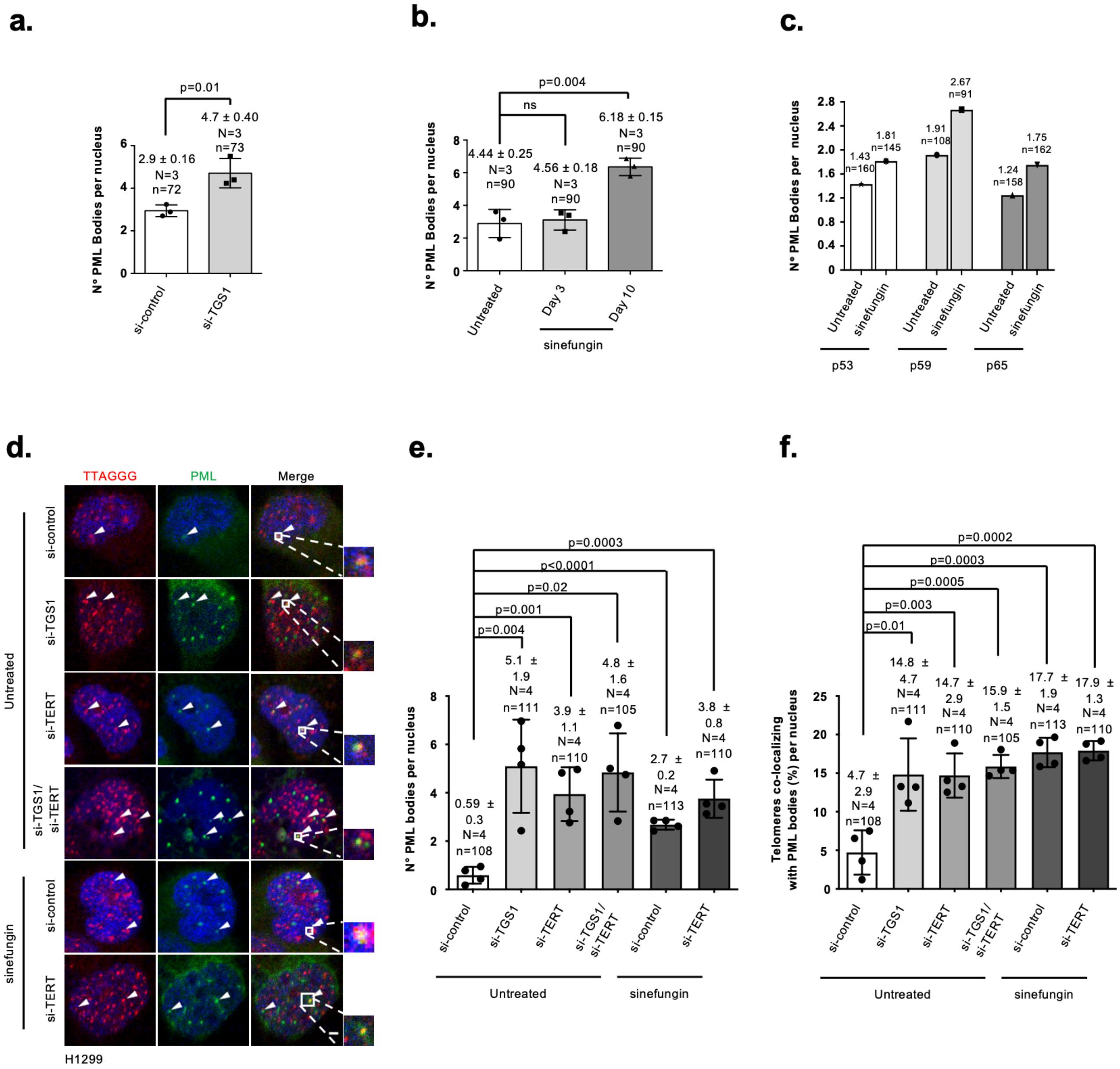

Supplementary Figure 5. Loss of TGS1 function promotes localization of telomeres to PML bodies.
a. Number of PML bodies per nucleus in H1299 cells repeatedly transfected for 10 days with the indicated siRNAs. **b.** Number of PML bodies per nucleus in H1299 cells untreated or treated with sinefungin for 3 and 10 days. **c.** Number of PML bodies per nucleus in lung tumor organoids untreated or treated with sinefungin for 10 days. **d.** Representative images of telomere DNA-FISH combined with immunofluorescence using anti-PML specific antibody in H1299 cells, repeatedly transfected with indicated siRNAs. When indicated, cells were treated with sinefungin. Scale bar 1 µm. **e.** Number of PML bodies per nucleus of cells shown in Suppl. Fig. 5d. **f.** Quantification of PML bodies co-localizing with telomeres (DNA-FISH) of cells shown in Suppl. Fig. 5d.
For quantifications in a, b, c, e and f mean values are indicated; whiskers indicate standard deviation. N=number of independent experiments. n=number of analyzed nuclei. Arrowheads indicate co-localization events. Red and green, epitopes detected by immunofluorescence; blue, DAPI stained nuclei. A two-tailed Student's t-test was used to calculate statistical significance; p-values are shown.

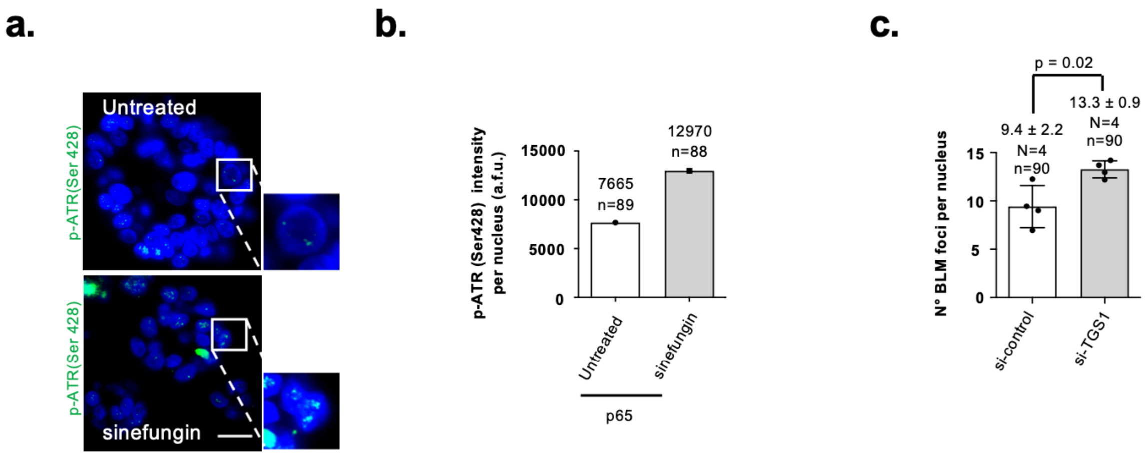

Supplementary Figure 6. Sinefungin treatment leads to the activation of ATR in lung tumor organoids.
a. Representative images of immunofluorescence using anti-phosphoATR specific antibody (phospho S428) on sections of lung tumor organoids untreated or treated with sinefungin for 10 days. Scale bar 10 µm. **b.** Quantification of phophoATR (S428) intensity per nucleus of cells shown in Suppl. Fig. 6a. **c.** Number of BLM foci per nucleus in H1299 cells repeatedly transfected for 10 days with the indicated siRNAs. For quantification in panel c, mean values are indicated; whiskers indicate standard deviation. N=number of independent experiments, n=number of analyzed nuclei. Green, epitope detected by immunofluorescence; blue, DAPI stained nuclei. A two-tailed Student's t-test was used to calculate statistical significance; p-values are shown.

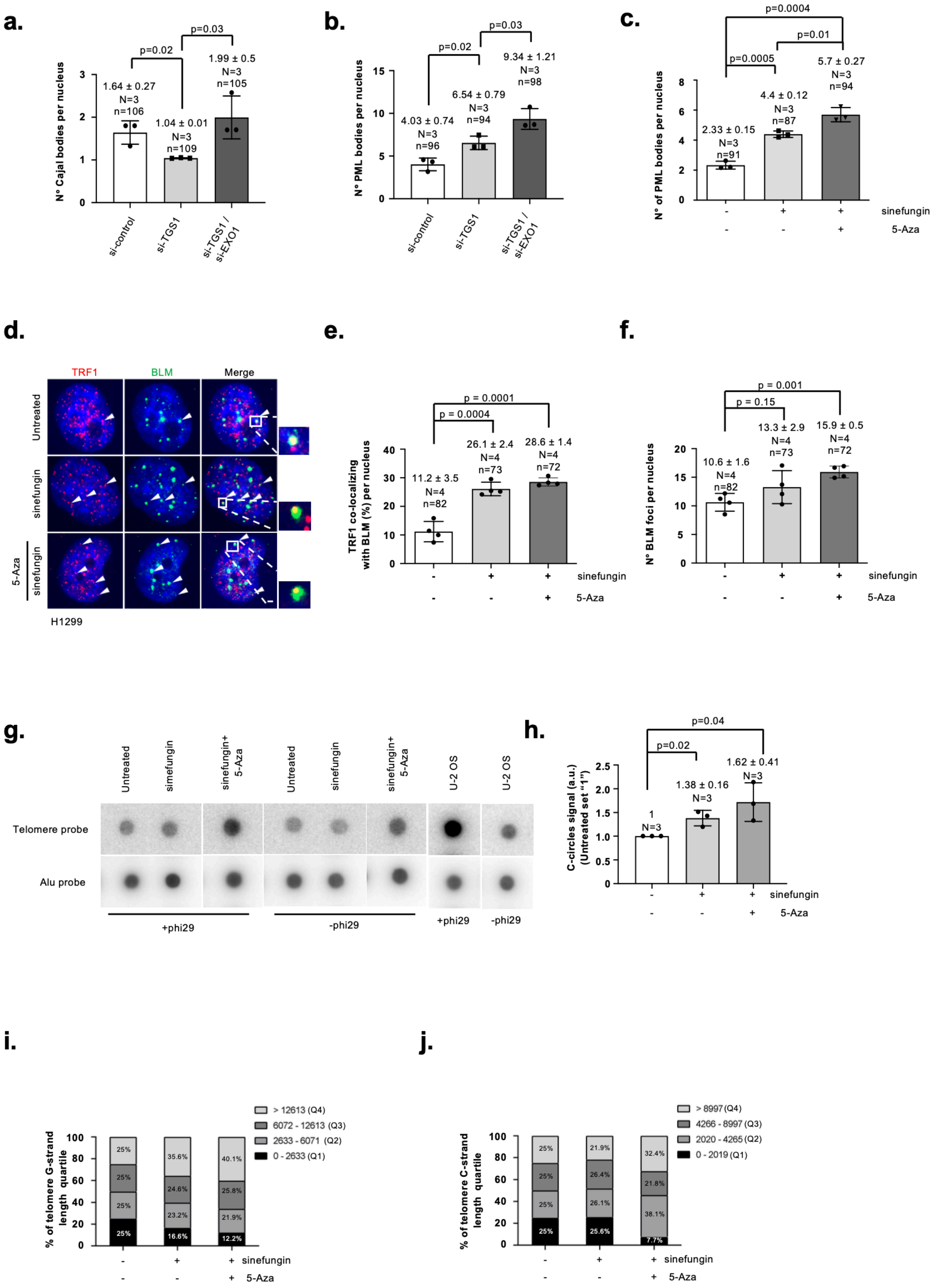

Supplementary Figure 7. Loss of TGS1 function mediates activation of ALT features.
a. Number of Cajal bodies per nucleus in H1299 cells repeatedly transfected for 10 days with the indicated siRNAs. **b**. Number of PML bodies per nucleus in H1299 cells repeatedly transfected for 10 days with the indicated siRNAs. **c.** Number of PML bodies per nucleus in H1299 cells untreated or treated with the indicated combination of drugs. **d.** Representative images of combined immunofluorescence with anti-TRF1 and anti-

BLM specific antibodies on H1299 cells untreated or treated with sinefungin and in combination with 5-Aza-2'-Deoxycytidine (5-Aza) for 10 days. Scale bar 1 µm. **e.** Percentage of BLM signals co-localizing with TRF1 per nucleus of experiment shown in Suppl. Fig. 7d. **f.** Number of BLM foci per nucleus in cells shown in Suppl. Fig. 7d. **g.** C-circle assay in H1299 cells untreated or treated with sinefungin and in combination with 5-Aza for 10 days. No phi29 and U-2 OS cells were used as negative and positive controls, respectively. Membranes were probed with a radiolabeled telomere probe; a human Alu repeat probe was used as loading control. **h.** Quantification of C-circles assays shown in Suppl. Fig. 7g. **i**. Representation of G-rich telomere strand FISH signals of samples described in Fig. 6k, l grouped into 4 quartiles, according to arbitrary telomere fluorescence units (a.f.u). Q1, quartile with shortest telomeres; Q4, quartile with longest telomeres (top 25%). **j.** Representation of C-rich telomere strand FISH signals of samples described in Fig. 6m, n grouped into 4 quartiles, according to arbitrary telomere fluorescence units (a.f.u). Q1, quartile with shortest telomeres; Q4, quartile with longest telomeres (top 25%). For quantifications in a, b, c, e, f, h mean values are indicated; whiskers indicate standard deviation. N=number of independent experiments. n=number of analyzed nuclei. Arrowheads indicate co-localization events. Red and green, epitopes detected by immunofluorescence; blue, DAPI stained nuclei. A two-tailed Student's t-test was used to calculate statistical significance; p-values are shown. Uncropped blots in Source Data.

Target	Company	Cod/sequence
Human TGS1	Dharmacon	ON-TARGET plus smartpool (FE5L017151000005)
Human RAD51	MWG EUROFINS	5’- GCAGUGAUGUCCUGGAUAA(TT) - 3'
Human EXO1	MWG EUROFINS	5'- CGUAAAUAGAAGAAUAAUU(TT) - 3'
Non-targeting control	Dharmacon	ON-TARGET plus Non-targeting siRNA #1(D-001810-01-05)

Supplementary table 1. Specific siRNA oligonucleotides used in this study.

Antibody	Company	WB	IF	IHC	ChIP/RIP/DRIP
Mouse anti–Actin	Sigma, A2228	1: 20000			
Rabbit anti-TGS1	Bethyl, A300-814A	1:500			
Mouse anti-2,2,7 trimethylguanosine clone K121	Millipore, MABE302				5 µg
Mouse anti-GAPDH (6C5)	Santa Cruz, sc-32233	1:5000			
Rabbit anti-Cytokeratin 7 (SP52)	Roche, 790-4462			1:200	
Mouse anti-Tubulin	Sigma, T5168	1:5000			
Mouse anti-TRF1 (TRF-78)	Abcam, ab10579	1:500			
Rabbit anti-TRF1(N-19)	Santa Cruz, sc-6165-R		1:100		
Rabbit anti-RAD51(H-92)	Santa Cruz, sc-8349		1:200		
Mouse anti-PML	Santa Cruz, sc-		1:100		
Mouse anti-TRF2(4A794)	Millipore, 05-521	1:500	1:200		2.5 µg
Mouse anti-Flag-tagM2 (cloneM2)	Sigma, F3165	1:1000	1:100		2.5 µg
Goat anti-BLM	Santa Cruz, sc-7790		1:100		
Mouse anti-DNA-RNA Hybrid [S9.6]	Kerafast, ENH001		1:100		3 µg
Rabbit anti-Coilin	Santa Cruz, sc-32860		1:100		
Rabbit anti-pATR(S428)	Cell Signaling, 2853s		1:100		
Mouse anti-POLD3	Novus Biologicals, H00010714- M01		1:100		
Rabbit anti-PML (H-238)	Santa Cruz, sc-5621		1:100		
Normal mouse IgG	SantaCruz, sc-2025				2.5 µg
Normal rabbit IgG	SantaCruz, sc-2027				2.5 µg
Goat anti-rabbit Alexafluor 488	Invitrogen		1:500		
Goat anti-mouse Alexafluor 488	Invitrogen		1:500		
Goat anti-rabbit Alexafluor 555	Invitrogen		1:500		
Goat anti-mouse Alexafluor 555	Invitrogen		1:500		

Supplementary table 2. Primary and secondary antibodies used in this study.

Oligo name	Sequence
hTERT FW	AACAAGCTGTTTGCGGGGAT
hTERT REV	CCAGGGTCCTGAGGAAGGTTT
β-ACTIN FW	AGCACTGTGTTGGCGTACAG
β-ACTIN REV	TCCCTGGAGAAGAGCTACGA
hTGS1 FW	AGGAGCGGAGGATTGTAAG
hTGS1 REV	TCCTCTTCTTCTGTCGCCTG
hTR FW	CCTAACTGAGAAGGGCGTAGG
hTR REV	GAATGAACGGTGGAAGGCG
hTRF1 FW	GCTGTTTGTATGGAAAATGGC
hTRF1 REV	CCGCTGCCTTCATTAGAAAG
hTRF2 FW	CATGCAGGCTTTGCTTGTCA
hTRF2 REV	CTGCATAACCCGGAGCAATA
GAPDH FW	CTGGTAAAGTGGATATTGTTGCCAT
GAPDH REV	TGGAATCATATTGGAACATGTAAACC

Supplementary table 3. PCR Oligonucleotides used in this study.
