# Supplementary material for: TGS1 mediates 2,2,7-trimethyl guanosine capping of the human telomerase RNA to direct telomerase dependent telomere maintenance
Source: Nat Commun. 2022 Apr 28;13:2302. doi: 10.1038/s41467-022-29907-z (PMC9050681; doi:10.1038/s41467-022-29907-z)
Supplement: Supplementary file 3 — Reporting Summary [file 41467_2022_29907_MOESM3_ESM.pdf]

nature portfolio

Corresponding author(s): Stefan Schoeftner

Last updated by author(s): Sep 22, 2021

Reporting Summary

Nature Portfolio wishes to improve the reproducibility of the work that we publish. This form provides structure for consistency and transparency in reporting. For further information on Nature Portfolio policies, see our Editorial Policies and the Editorial Policy Checklist.

Statistics

For all statistical analyses, confirm that the following items are present in the figure legend, table legend, main text, or Methods section.

n/a	Confirmed	
☐	☒	The exact sample size (*n*) for each experimental group/condition, given as a discrete number and unit of measurement
☐	☒	A statement on whether measurements were taken from distinct samples or whether the same sample was measured repeatedly
☐	☒	The statistical test(s) used AND whether they are one- or two-sided<br>*Only common tests should be described solely by name; describe more complex techniques in the Methods section.*
☒	☐	A description of all covariates tested
☒	☐	A description of any assumptions or corrections, such as tests of normality and adjustment for multiple comparisons
☐	☒	A full description of the statistical parameters including central tendency (e.g. means) or other basic estimates (e.g. regression coefficient) AND variation (e.g. standard deviation) or associated estimates of uncertainty (e.g. confidence intervals)
☐	☒	For null hypothesis testing, the test statistic (e.g. *F*, *t*, *r*) with confidence intervals, effect sizes, degrees of freedom and *P* value noted<br>*Give P values as exact values whenever suitable.*
☒	☐	For Bayesian analysis, information on the choice of priors and Markov chain Monte Carlo settings
☒	☐	For hierarchical and complex designs, identification of the appropriate level for tests and full reporting of outcomes
☒	☐	Estimates of effect sizes (e.g. Cohen's *d*, Pearson's *r*), indicating how they were calculated

Our web collection on statistics for biologists contains articles on many of the points above.

Software and code

Policy information about availability of computer code

Data collection	Cells subjected to immunofluorescence, RNA FISH, immunofluorescence combined with RNA FISH, CO-FISH or telomere DNA FISH were captured using Leica Application Suite (LAS) imaging software. For confocal acquisition of immunostained cells a NIS elements 5.2 imaging software (Nikon) was used. For immunohistochemistry acquisition a D-sight digital microscope was used. For c-circles assay and G-strand overhang assay Typhoon Imager (GE Healthcare, Chicago, IL, USA) was used.
Data analysis	ImageJ v.1.53e, Fiji v.1.52e, TFL-TELO v.2 and Adobe Photoshop C6 for image analysis and processing; Microsoft Excel and Graph Pad Prism 6 for statistical analysis and graph plotting.<br>Exome sequencing: Quality checks for raw reads were performed using FastQC (http://www.bioinformatics.babraham.ac.uk/projects/fastqc). High-quality reads were aligned to the GRCh37 human reference genome by BWA (1) after adapter and PCR duplicate removal by the Trimmomatic (2) and MarkDuplicates module from Picard tools (Broad Institute: Picard:A set of command line tools (in Java) for manipulating high-throughput sequencing (HTS) data and formats such as SAM/BAM/CRAM and VCF. http://broadinstitute.github.io/picard/), respectively. De-duplicated reads were realigned at known indel positions and systematic sequencing errors fixed with recalibration by GATK (https://software.broadinstitute.org/gatk/best-practices). The somatic single nucleotide variants (SNVs) and short indels were identified in tumor tissues and organoids versus the matched normal tissues by integrating MuTect2 (3) and VarScan2 (4) algorithms, with default parameters. Somatic variants were annotated using Annovar (5).<br>(1). Li, H. & Durbin, R. Fast and accurate short read alignment with Burrows-Wheeler transform. Bioinformatics (2009). doi:10.1093/bioinformatics/btp324<br>(2). Bolger, A. M., Lohse, M. & Usadel, B. Trimmomatic: A flexible trimmer for Illumina sequence data. Bioinformatics (2014). doi:10.1093/bioinformatics/btu170<br>(3). Cibulskis, K. et al. Sensitive detection of somatic point mutations in impure and heterogeneous cancer samples. Nat. Biotechnol. 31, 213–219 (2013).<br>(4). Koboldt, D. C., Larson, D. E. & Wilson, R. K. Using varscan 2 for germline variant calling and somatic mutation detection. Curr. Protoc. Bioinforma. (2013). doi:10.1002/0471250953.bi1504s44

(5). Wang, K., Li, M. & Hakonarson, H. ANNOVAR: Functional annotation of genetic variants from high-throughput sequencing data. Nucleic Acids Res. 38, (2010).

For manuscripts utilizing custom algorithms or software that are central to the research but not yet described in published literature, software must be made available to editors and reviewers. We strongly encourage code deposition in a community repository (e.g. GitHub). See the Nature Portfolio guidelines for submitting code & software for further information.

Data

Policy information about availability of data

All manuscripts must include a data availability statement. This statement should provide the following information, where applicable:

- Accession codes, unique identifiers, or web links for publicly available datasets
- A description of any restrictions on data availability
- For clinical datasets or third party data, please ensure that the statement adheres to our policy

Due to patient privacy reasons, exome sequencing data are available only under restricted access. Upon reasonable request, access can be obtained by contacting the corresponding author.

Field-specific reporting

Please select the one below that is the best fit for your research. If you are not sure, read the appropriate sections before making your selection.

☒ Life sciences ☐ Behavioural & social sciences ☐ Ecological, evolutionary & environmental sciences

For a reference copy of the document with all sections, see nature.com/documents/nr-reporting-summary-flat.pdf

Life sciences study design

All studies must disclose on these points even when the disclosure is negative.

Sample size	Sample sizes were chosen according to accepted standards in the field. Sample size was not pre-determined using statistics tolls. Minimal size of analyzed biological samples was "3". Data from tumor organoids were obtained from 2 or 3 different patients. Details on sample numbers can be obtained from the respective figures and figure legends. Statistical analysis (as described in respective figure legends) was used to calculate statistical significance of obtained results. The individual p-values are indicated in all figures.
Data exclusions	No data exclusion
Replication	All experiments were repeated a minimum of three times. Data from tumor organoids were obtained from 2 or 3 different patients. We specified the number of biological replicates in the respective figures.
Randomization	Pictures of fluorescence, H&E or IHC stained biological samples were randomly taken; All experiments are based on gain or loss of function experiments with appropriate controls and were repeated at least 3 times.
Blinding	No blinding was applied. Multiple members of the research team obtained same result from individual experiments.

Reporting for specific materials, systems and methods

We require information from authors about some types of materials, experimental systems and methods used in many studies. Here, indicate whether each material, system or method listed is relevant to your study. If you are not sure if a list item applies to your research, read the appropriate section before selecting a response.

Materials & experimental systems

n/a	Involved in the study	
☐	☒	Antibodies
☐	☒	Eukaryotic cell lines
☒	☐	Palaeontology and archaeology
☒	☐	Animals and other organisms
☐	☒	Human research participants
☒	☐	Clinical data
☐	☒	Dual use research of concern

Methods

n/a	Involved in the study	
☒	☐	ChIP-seq
☒	☐	Flow cytometry
☒	☐	MRI-based neuroimaging

Antibodies

Antibodies used	Mouse anti–Actin (Sigma, A2228)

Rabbit anti-TGS1 (Bethyl, A300-814A)
Mouse anti-2,2,7 trimethylguanosine clone K121 (Millipore, MABE302)
Mouse anti-GAPDH (6C5) (Santa Cruz, sc-32233)
Rabbit anti-Cytokeratin 7 (SP52) (Roche, 790-4462)
Mouse anti-Tubulin (Sigma, T5168)
Mouse anti-TRF1 (TRF-78) (Abcam, ab10579)
Rabbit anti-TRF1(N-19) (Santa Cruz, sc-6165-R)
Rabbit anti-RAD51(H-92) (Santa Cruz, sc-8349)
Mouse anti-PML (Santa Cruz, sc-377390)
Mouse anti-TRF2(4A794) (Millipore, 05-521)
Mouse anti-Flag-tagM2 (cloneM2) (Sigma, F3165)
Goat anti-BLM (Santa Cruz, sc-7790)
Mouse anti-DNA-RNA Hybrid [S9.6] (Kerafast, ENH001)
Rabbit anti-Coilin (Santa Cruz, sc-32860)
Rabbit anti-pATR(S428) (Cell Signaling, 2853s)
Mouse anti-POLD3 (Novus Biologicals, H00010714- M01)
Rabbit anti-PML (H-238) (Santa Cruz, sc-5621)
Normal mouse IgG (SantaCruz, sc-2025)
Normal rabbit IgG (SantaCruz, sc-2027)
Goat anti-rabbit Alexafluor 488 (Invitrogen)
Goat anti-mouse Alexafluor 488 (Invitrogen)
Goat anti-rabbit Alexafluor 555 (Invitrogen)
Goat anti-mouse Alexafluor 555 (Invitrogen)

Validation

The specificity of rabbit anti-TGS1 was confirmed by western blot analysis of siRNA-depleted cells (Fig.1 A). The specificity of Rabbit anti-Coilin was previously validated by Stern et al. "Telomerase recruitment requires both TCAB1 and Cajal bodies independently" (Mol Cell Biol. 2012 Jul;32(13):2384-95. doi: 10.1128/MCB.00379-12). Rabbit anti-TRF1 antibody was previously validated by Petti et al. "SFPQ and NONO suppress RNA:DNA-hybrid-related telomere instability" (Nat Commun. 2019 Mar 1;10(1):1001. doi: 10.1038/s41467-019-08863-1). Mouse anti-Flag specificity was confirmed by Western Blot analysis of cells over-expressing Flag-tagged protein (Suppl. Fig. 2D). The specificity of mouse anti-TRF2 antibody was previously validated by Petti et al. "SFPQ and NONO suppress RNA:DNA-hybrid-related telomere instability" (Nat Commun. 2019 Mar 1;10(1):1001. doi: 10.1038/s41467-019-08863-1). The specificity of rabbit anti-PML was previously validated by Ahmed, A. et al. "Regulation of NF-κB by PML and PML-RARα." (Sci. Rep. 7, 44539; doi: 10.1038/srep44539 (2017)). The specificity of the anti-DNA:RNA hybrids S9.6 antibody was confirmed by indirect immunofluorescence staining performed in cells treated with RNase H (Fig. 5A). Rabbit anti-phospho-ATR antibody specificity has been stated on the manufacturer's website (Cell Signalling technology). The specificity of rabbit anti-RAD51 antibody was previously validated by Serrano, M.A. et al. "DNA-PK, ATM and ATR collaboratively regulate p53-RPA interaction to facilitate homologous recombination DNA repair." (Oncogene. 2013 May 9; 32(19): 2452–2462. doi:10.1038/onc.2012.257). Goat- anti BLM antibody was previously validated by Passerini et al "The presence of extra chromosomes leads to genomic instability" (Nat Commun. 2016 Feb 15;7:10754. doi: 10.1038/ncomms10754). Mouse anti-POLD3 antibody specificity has been stated on the manufacturer's website (Novus Biologicals). Rabbit anti-coilin antibody specificity is stated on the manufacturer's website (SantaCrutz).
The specificity of mouse anti-2,2,7 trimethylguanosine clone K121 antibody was confirmed by RNA immunoprecipitation experiment, showing a specific interaction with trimethylguanosine-capped RNA but not with monomethilguanosine-capped RNA (Suppl. fig. 3A-C). The specificity of Rabbit anti-Cytokeratin 7 (SP52) is stated on the manufacturer's website (ThermoFisher Scientific).
The specificity of mouse anti-PML was previously validated by Sidik et al. "Shigella infection interferes with SUMOylation and increases PML-NB number" (PLoS One. 2015 Apr 7;10(4):e0122585. doi: 10.1371/journal.pone.0122585. eCollection 2015).

Normal mouse IgG was used as negative control as shown in Fig. 2M.

Eukaryotic cell lines

Policy information about cell lines

Cell line source(s)

U-2 OS and H1299 cells were obtained by ATCC

Authentication

Cells were obtained by ATCC; characteristic features such as telomere length, telomere length distribution, the presence of APBs and metaphase chromosome characteristics was experimentally validated

Mycoplasma contamination

All cell lines used in the study were tested negative by DAPI staining and PCR analysis for mycoplasma contamination

Commonly misidentified lines
(See ICLAC register)

No commonly misidentified lines were used in the study

Human research participants

Policy information about studies involving human research participants

Population characteristics

Criteria for patient enrolment to the observational study:
Age: ≥ 18 years
Obtained informed consent from all participants and this is noted in the manuscript.
Dimension of lesion: ≥1cm.
Patients recruitied in the study did not show documented infectious disease, metastasis and did not receive neoadjuvant therapy

Recruitment	All patients described in this study were enrolled into the observational stady carried out at the Azienda Sanitaria Universitaria Giuliano Isontina (ASUGI).<br>Patients were selected by:<br>1. F.Z; co-author of this study; Head of Pathology Unit, Struttura Complessa di Anatomia ed Istologia Patologica, Azienda Sanitaria Universitaria Giuliano Isontina (ASUGI)<br>2. M.C; co-author of this study; Head of the Thoracic Surgery Unit. Struttura Complessa di Chirurgia Toracica, Azienda Sanitaria Universitaria Giuliano Isontina (ASUGI) Trieste
Ethics oversight	The research protocol was approved by the ethics committee of the region Friuli Venezia Giulia, Italy (comitato etico unico regionale, prot. n. 5798, 22.02.2018, Aviano, Italy)

Note that full information on the approval of the study protocol must also be provided in the manuscript.

Dual use research of concern

Policy information about dual use research of concern

Hazards

Could the accidental, deliberate or reckless misuse of agents or technologies generated in the work, or the application of information presented in the manuscript, pose a threat to:

No	Yes	
☒	☐	Public health
☒	☐	National security
☒	☐	Crops and/or livestock
☒	☐	Ecosystems
☒	☐	Any other significant area

Experiments of concern

Does the work involve any of these experiments of concern:

No	Yes	
☒	☐	Demonstrate how to render a vaccine ineffective
☒	☐	Confer resistance to therapeutically useful antibiotics or antiviral agents
☒	☐	Enhance the virulence of a pathogen or render a nonpathogen virulent
☒	☐	Increase transmissibility of a pathogen
☒	☐	Alter the host range of a pathogen
☒	☐	Enable evasion of diagnostic/detection modalities
☒	☐	Enable the weaponization of a biological agent or toxin
☒	☐	Any other potentially harmful combination of experiments and agents
